# Supplementary material for: Evolving brain function and connectivity patterns during mentalizing in children and adults
Source: Commun Biol. 2026 Jan 21;9:282. doi: 10.1038/s42003-026-09562-6 (PMC12920907; doi:10.1038/s42003-026-09562-6)
Supplement: Supplementary file 2 — Supplemental Information [file 42003_2026_9562_MOESM2_ESM.pdf]

Supplementary material to:

Evolving Brain Function and Connectivity during Mentalizing in Children and Adults

Supplementary Figure 1. Age-dependent mentalizing performance in children

Association between age (in years; x-axis) and overall mentalizing performance in children.

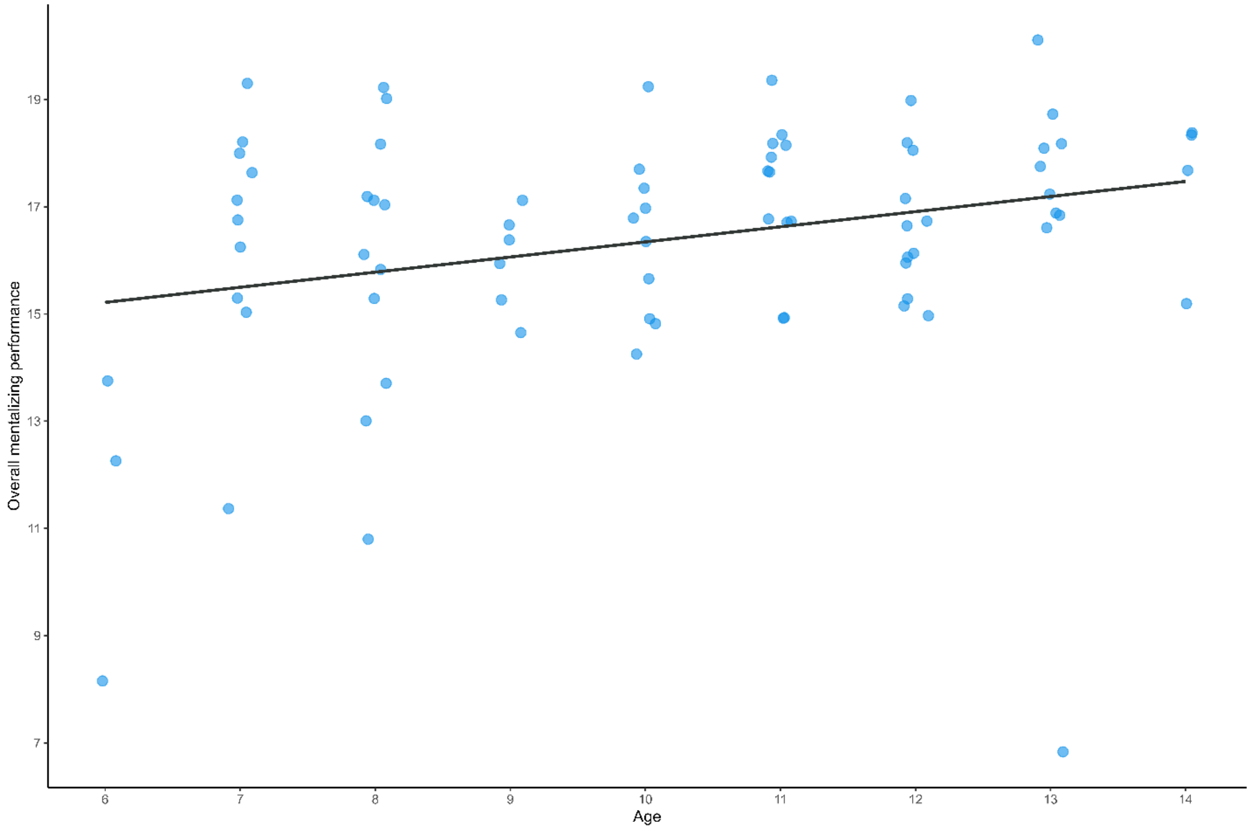

<u>**Supplementary Figure 2.** Further fMRI task specifications (CAToon task)</u>

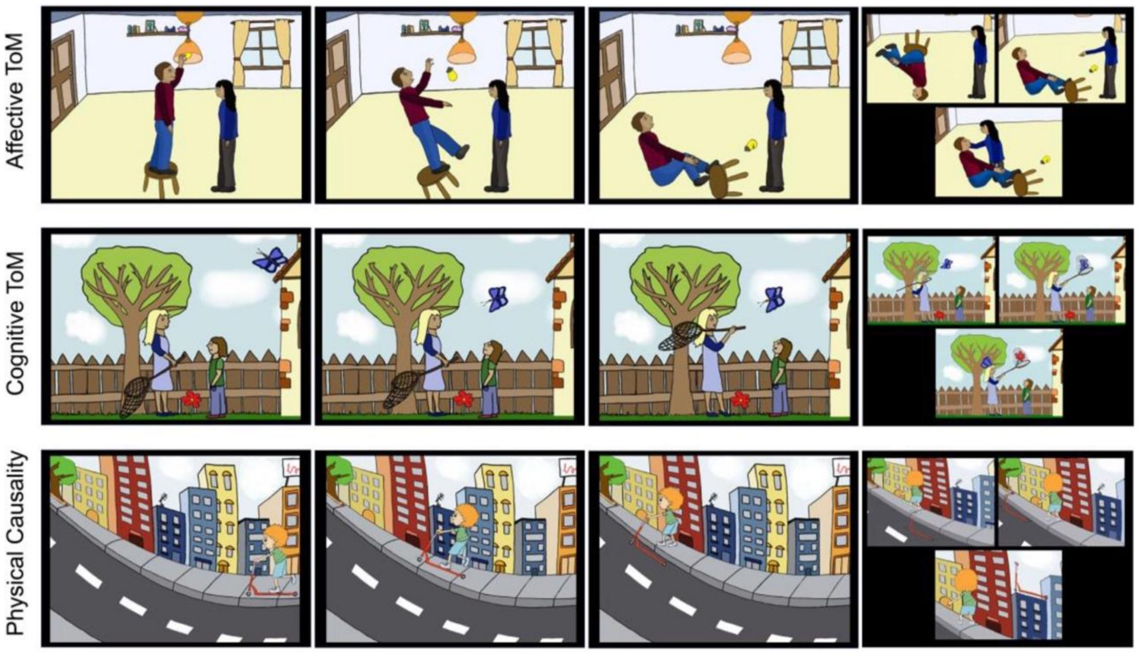

Figure illustrating task design. Adapted from (Borbás et al., 2021). One example trial for each condition of the CAToon task.

<u>**Supplementary Figure 3.** ROI selection / fMRI alignment</u>

Overlap of functional activation during mentalizing in children and adults (in orange and red, respectively) and of the selected regions of interest (ROIs) based on Dufour 2016 (in blue).

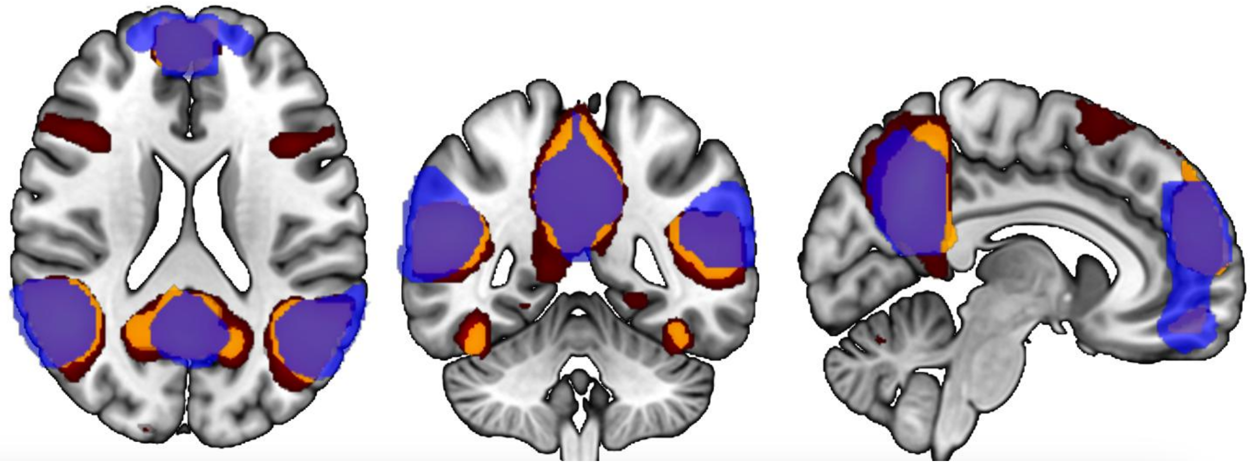

Supplementary Method 1. Task presentation in the fMRI

Stimuli were shown on a screen positioned behind the scanner and viewed by participants via a prism attached to the head coil and displayed using Presentation® software (V16.5, Neurobehavioral Systems, Inc., Berkeley, CA, www.neurobs.com). The trial order was once pseudo randomized (see (Borbás et al., 2021)), but remained constant for all participants. While 59 adults completed the task in one run. The task was kept identical but split into two runs with a short break in between for children (N=80) in order to reduce task demands. 42 adults performed the same version of the task, divided into two runs. Based on pilot feedback from a subset of young adult participants, the original 7-second response window was found to be challenging for some individuals. To ensure the task remained accessible and developmentally appropriate for children, the response window was extended to 10 seconds. For consistency across age groups, this adjustment was also applied in subsequent sessions with adult participants.

Supplementary Method 2. Details on fMRI image acquisition

Site 1 (Basel). **Functional.** For the T2*-weighted echo-planar images transverse slice orientation, interleaved acquisition was used, and the following specifics: field of view = 220 mm, flip angle = 83 degrees, TR = 2000 ms, TE = 30 ms, 42 slices, slice thickness = 2 mm, voxel size = 2.0 × 2.0 × 2.0 mm. **Structural.** Whole-brain structural MPRAGE images were acquired on a SIEMENS 3T Prisma MR scanners using a 20-channel head coil using the following specifications: Voxel size: 1.0 × 1.0 × 1.0 mm^3; TR= 1900 ms; TE= 3.42 ms TA= 4.26 min; flip angle= 9 degrees; field of view= 256×256mm2, 192 slices with a slice thickness of 1.00 mm. The acquisition lasted 4 min and 26 s.

Site 2 (Zürich). **Functional.** For acquiring the T2*-weighted echo-planar images following specifications were used: field of view = 240 mm, flip angle = 83 degrees, TR = 2200 ms, TE = Min Full, 41 slices, slice thickness = 2 mm, voxel size = 2.0 × 2.0 × 2.0 mm. **Structural.** T1-weighted data was acquired on a General Electric 3T scanner equipping a 48-channel head coil. Following specifics were applied: voxel size: 1.0 × 1.0 × 1.0 mm; TR= 8.2 ms; TE= 3.4 ms; TA= 3.22; flip angle= 12 degrees; field of view= 256×256mm, 176 slices with a slice thickness of 1.00 mm.

Supplementary Method 3. Number of scans excluded from analyses

Children (mean number of exclusion: 1.05) and adults (mean number of exclusions: 0.74) did not significantly differ in the number of scans excluded (p=0.604). Of all images included in the analyses the number of scans excluded in the children's group ranged from 0-4.6% (14 images of 304 total images acquired) images and from 0-3.0% (9 images of 304 total images acquired) images in adults.
