# Supplementary material for: Evolving brain function and connectivity patterns during mentalizing in children and adults
Source: Commun Biol. 2026 Jan 21;9:282. doi: 10.1038/s42003-026-09562-6 (PMC12920907; doi:10.1038/s42003-026-09562-6)
Supplement: Supplementary file 3 — Description of Additional Supplementary Materials [file 42003_2026_9562_MOESM3_ESM.pdf]

Description of Additional Supplementary Files

File name: Supplementary Data

Description: The raw data behind the graphs in Figure 3A
