# Supplementary material for: Evolving brain function and connectivity patterns during mentalizing in children and adults
Source: Commun Biol. 2026 Jan 21;9:282. doi: 10.1038/s42003-026-09562-6 (PMC12920907; doi:10.1038/s42003-026-09562-6)
Supplement: Supplementary file 5 — Reporting Summary [file 42003_2026_9562_MOESM5_ESM.pdf]

nature portfolio

Corresponding author(s): Nora Maria Raschle
Last updated by author(s): Dec 10, 2025

Reporting Summary

Nature Portfolio wishes to improve the reproducibility of the work that we publish. This form provides structure for consistency and transparency in reporting. For further information on Nature Portfolio policies, see our Editorial Policies and the Editorial Policy Checklist.

Statistics

For all statistical analyses, confirm that the following items are present in the figure legend, table legend, main text, or Methods section.

n/a	Confirmed	
☐	☒	The exact sample size (*n*) for each experimental group/condition, given as a discrete number and unit of measurement
☐	☒	A statement on whether measurements were taken from distinct samples or whether the same sample was measured repeatedly
☐	☒	The statistical test(s) used AND whether they are one- or two-sided<br>*Only common tests should be described solely by name; describe more complex techniques in the Methods section.*
☐	☒	A description of all covariates tested
☐	☒	A description of any assumptions or corrections, such as tests of normality and adjustment for multiple comparisons
☐	☒	A full description of the statistical parameters including central tendency (e.g. means) or other basic estimates (e.g. regression coefficient) AND variation (e.g. standard deviation) or associated estimates of uncertainty (e.g. confidence intervals)
☐	☒	For null hypothesis testing, the test statistic (e.g. *F*, *t*, *r*) with confidence intervals, effect sizes, degrees of freedom and *P* value noted<br>*Give P values as exact values whenever suitable.*
☒	☐	For Bayesian analysis, information on the choice of priors and Markov chain Monte Carlo settings
☒	☐	For hierarchical and complex designs, identification of the appropriate level for tests and full reporting of outcomes
☐	☒	Estimates of effect sizes (e.g. Cohen's *d*, Pearson's *r*), indicating how they were calculated

Our web collection on statistics for biologists contains articles on many of the points above.

Software and code

Policy information about availability of computer code

Data collection	SIEMENS 3T Prisma MR scanner and 3T GE Signa Premier MRI scanner, Paperbased, Limesurvey (https://www.limesurvey.org; hosted at UZH), Presentation® software (V16.5, Neurobehavioral Systems, Inc., Berkeley, CA, www.neurobs.com)
Data analysis	Analyses were conducted in SPM ([54]; RRID:SCR-007037; release 12.7771; in MATLAB (R2023a)), R Studio, CONN toolbox (release 22.a), SPSS (IBM Corp. Released 2017. IBM SPSS Statistics for Windows, Version 27.0. Armonk, NY: IBM Corp.))

For manuscripts utilizing custom algorithms or software that are central to the research but not yet described in published literature, software must be made available to editors and reviewers. We strongly encourage code deposition in a community repository (e.g. GitHub). See the Nature Portfolio guidelines for submitting code & software for further information.

Data

Policy information about availability of data

All manuscripts must include a data availability statement. This statement should provide the following information, where applicable:

- Accession codes, unique identifiers, or web links for publicly available datasets
- A description of any restrictions on data availability
- For clinical datasets or third party data, please ensure that the statement adheres to our policy

All data relevant to the current manuscript are available in accordance with the restrictions specified by the corresponding ethical agreements. Due to the

vulnerability of young participants, only de-identified data are shared. This may include selected behavioral data points. For neuroimaging data, extracted scores, group-level maps, and findings have been made available via NeuroVault

Research involving human participants, their data, or biological material

Policy information about studies with human participants or human data. See also policy information about sex, gender (identity/presentation), and sexual orientation and race, ethnicity and racism.

Reporting on sex and gender	Recruitement included participants of both sex. Sex is reported and accounted for in all analyses as a covariate of no interest.
Reporting on race, ethnicity, or other socially relevant groupings	Since IQ scores were only available for some adults, we additionally calculated their highest level of education, assessed using the International Standard Classification of Education (ISCED, 2011). The average educational attainment was ISCED level 5.34±2.05, indicating that most had completed tertiary education levels.<br><br>Eurostat and U.I.f. Statistics, ISCED 2011 Operational Manual Guidelines for Classifying National Education Programmes and Related Qualifications: Guidelines for Classifying National Education Programmes and Related Qualifications. 2015: OECD publishing.
Population characteristics	Mental well-being of all participants was confirmed to be in the average range using the Strengths and Difficulties Questionnaire for children and the Brief Symptom Inventory for adults.
Recruitment	Participants were recruited via social media, flyers, online platforms, and word-of-mouth and tested at two sites in Switzerland (Basel and Zurich). 80 children (29 females, 51 males; mean age = 10.1 years, range: 6-14 years) and 101 adults were included in the analyses (71 female, 30 males; mean age = 38.65 years, range: 20-61 years). Participants had sufficient German proficiency to understand the fMRI task instructions and an average or above-average IQ.
Ethics oversight	The study was approved by the Ethics Committee Northwest and Central Switzerland (Basel) and the Cantonal Ethics Committee Zurich. Adult participants provided written consent, while parental written consent and child assent were obtained for children.

Note that full information on the approval of the study protocol must also be provided in the manuscript.

Field-specific reporting

Please select the one below that is the best fit for your research. If you are not sure, read the appropriate sections before making your selection.

☒ Life sciences ☐ Behavioural & social sciences ☐ Ecological, evolutionary & environmental sciences

For a reference copy of the document with all sections, see nature.com/documents/nr-reporting-summary-flat.pdf

Life sciences study design

All studies must disclose on these points even when the disclosure is negative.

Sample size	To determine the appropriate sample size for the main study, we used NeuroPowerTools, a power and sample size calculator for fMRI studies (Durnez et al., 2016). Power analyses based on Random Field Theory and conservative assumptions indicated that a power of at least 80% could be achieved with a sample size of N = 32 adults. For children, the same level of power required N = 55 participants. However, to model life-course trajectories, representation across childhood and across early, middle, or late stages of adulthood was necessary, thereby increasing the required sample size in accordance with the aims of the present study. The final sample included 80 children (29 females, 51 males; mean age = 10.1 years, range: 6–14 years) and 101 adults (71 females, 30 males; mean age = 38.65 years, range: 20–61 years).
Data exclusions	14 children and 14 adult datasets were not included in the analyses due to low data quality, braces, excessive movement, claustrophobia, incomplete tasks, developmental delay, or visual impairments.
Replication	All analyses yielded the same results when using the same tools, methods, and dataset. Whether these results replicate in independent samples remains to be tested in future research.
Randomization	No randomization was performed, as the study did not include experimental group assignments.
Blinding	Blinding was not applicable in this study, as no experimental conditions or group assignments were involved.

Reporting for specific materials, systems and methods

We require information from authors about some types of materials, experimental systems and methods used in many studies. Here, indicate whether each material, system or method listed is relevant to your study. If you are not sure if a list item applies to your research, read the appropriate section before selecting a response.

Materials & experimental systems

n/a	Involved in the study
☒	☐ Antibodies
☒	☐ Eukaryotic cell lines
☒	☐ Palaeontology and archaeology
☒	☐ Animals and other organisms
☒	☐ Clinical data
☒	☐ Dual use research of concern
☒	☐ Plants

Methods

n/a	Involved in the study
☒	☐ ChIP-seq
☒	☐ Flow cytometry
☐	☒ MRI-based neuroimaging

Plants

Seed stocks: NA

Novel plant genotypes: NA

Authentication: NA

Magnetic resonance imaging

Experimental design

Design type: Block design

Design specifications: There were 2 experimental conditions (affective theory of mind and cognitive theory of mind) and 1 control condition (physical causality) with 10 trials each, totalizing 30 trials. Mentalizing included trials during the affective and cognitive condition. While 59 adults completed the task in one run. The task was kept identical, but split into two runs with a short break in between for children (N=80) in order to reduce task demands. 42 adults performed the same version of the task, divided into two runs. Based on pilot feedback from a subset of young adult participants, the original 7-second response window was found to be challenging for some individuals. To ensure the task remained accessible and developmentally appropriate for children, the response window was extended to 10 seconds. For consistency across age groups, this adjustment was also applied in subsequent sessions with adult participants.

Behavioral performance measures: Button box press at every trial, represented by the overall accuracy and accuracy in mentalizing trials (percentage of correct answers).

Acquisition

Imaging type(s): Functional

Field strength: 3T

Sequence & imaging parameters: Site 1 (Basel). Functional. For the T2*-weighted echo-planar images transverse slice orientation, interleaved acquisition was used, and the following specifics: field of view = 220 mm, flip angle = 83 degrees, TR = 2000 ms, TE = 30 ms, 42 slices, slice thickness = 2 mm, voxel size = 2.0 × 2.0 × 2.0 mm. Structural. Whole-brain structural MPRAGE images were acquired on a SIEMENS 3T Prisma MR scanners using a 20-channel head coil using the following specifications: Voxel size: 1.0 × 1.0 × 1.0 mm3; TR= 1900 ms; TE= 3.42 ms TA= 4.26 min; flip angle= 9 degrees; field of view= 256×256mm2, 192 slices with a slice thickness of 1.00 mm. The acquisition lasted 4 min and 26 s.
Site 2 (Zürich). Functional. For acquiring the T2*-weighted echo-planar images following specifications were used: field of view = 240 mm, flip angle = 83 degrees, TR = 2200 ms, TE = Min Full, 41 slices, slice thickness = 2 mm, voxel size = 2.0 × 2.0 × 2.0 mm. Structural. T1-weighted data was acquired on a General Electric 3T scanner equipping a 48-channel head coil. Following specifics were applied: voxel size: 1.0 × 1.0 × 1.0 mm; TR= 8.2 ms; TE= 3.4 ms; TA= 3.22; flip angle= 12 degrees; field of view= 256×256mm, 176 slices with a slice thickness of 1.00 mm.

Area of acquisition: Whole-brain.

Diffusion MRI: ☐ Used ☒ Not used

Preprocessing

Preprocessing software	SPM (RRID:SCR-007037; release 12.7771; in MATLAB (R2023a)
Normalization	Standard normalization procedure from SPM was conducted during the preprocessing, which included standard steps, such as realignment to the first image, co-registration to each participant's structural image, normalization to standard space (Montreal Neurological Institute template) and smoothed using an 8-mm full width at half maximum isotropic kernel.
Normalization template	Montreal Neurological Institute template
Noise and artifact removal	Movement and signal outliers were detected using the ART toolbox. Movement outliers exceeding 2 mm were excluded from the analyses
Volume censoring	No volume censoring was applied.

Statistical modeling & inference

Model type and settings	Mass univariate analysis using general linear models were conducted. First-level analyses modeled task events as boxcar functions convolved with the canonical hemodynamic response function. Regressors of interest included the full trial durations for 'affective theory of mind', 'cognitive theory of mind', and 'physical causality' conditions, including decision time. Second level analyses included age, site, and sex as covariates.
Effect(s) tested	Mentalizing was based on 'affective theory of mind' and 'cognitive theory of mind' trials; the control condition for fMRI contrasts also included ('physical causality'). Whole brain fMRI data was assessed using t-tests.

Specify type of analysis: ☐ Whole brain ☐ ROI-based ☒ Both

Anatomical location(s): Activation maps were labeled using the automated anatomical labeling atlas [57].

Statistic type for inference (See Eklund et al. 2016)	Whole-brain FWE correction (voxel-level); p(FWE-corrected)<0.05
Correction	Whole-brain FWE correction (voxel-level); p(FWE-corrected)<0.05

Models & analysis

n/a	Involved in the study
☐	☒ Functional and/or effective connectivity
☒	☐ Graph analysis
☒	☐ Multivariate modeling or predictive analysis

Functional and/or effective connectivity

For functional connectivity, the CONN toolbox (release 22.a) was used. Anatomical data was normalized into standard MNI space and resampled to 1 mm isotropic voxels using SPM unified segmentation and normalization algorithm with the default IXI-549 tissue probability map template. The first level fMRI models including condition of interest (mentalizing) were imported into the CONN toolbox. The data were denoised including the regression of potential confounding effects characterized by white matter timeseries, CSF timeseries, SPM covariates regressors, motion parameters and their first order derivatives, ART covariates regressors, session and task effects and their first order derivatives, and linear trends within each functional run. Bandpass frequency filtering of the BOLD time series was applied between 0.008 Hz and 0.09 Hz. First-Level Inferences. ROI-to-ROI connectivity matrices were estimated to characterize functional connectivity between each pair of the seven a priori ROIs. Functional connectivity strength was quantified using Fisher-transformed bivariate correlation coefficients from a weighted general linear model (weighted-GLM, calculated for each pair of ROIs to assess the association between their BOLD signal time series. Second-Level Inferences. For both child and adult groups, a weighted-GLM approach was employed. A separate GLM was estimated for each individual connection, with first-level connectivity measures as dependent variables, and subjects as the independent variable, while accounting for age, sex, and site. Connection-level statistics were evaluated using multivariate parametric statistics with random effects across subjects and sample covariance estimation across multiple measurements. Inferences were made at the individual connection level, with results thresholded using familywise corrected p-FDR <0.05. Two-tailed one-sample t-tests were performed separately for the children and adults to test whether average functional connectivity between the seven regions of interest differed from zero during mentalizing, controlling for age, sex, and site. Age effects in children were plotted using a one-tailed bivariate regression analysis (based on the directed hypothesis of stronger and more extensive functional connectivity with increasing age). In adults, two two-tailed tests were conducted to account for possible in- or decreases with age in functional connectivity. COMBAT was used on extracted connectivity scores for site-harmonization.
